# Impact of lifestyle and mental health on colorectal adenomas in China: a prospective cross-sectional survey

**DOI:** 10.3389/fmed.2025.1475987

**Published:** 2025-03-03

**Authors:** Min Ye, Shiben Zhu, Xinyi Tan, Chenxi Yu, He Huang, Yang Liu

**Affiliations:** ^1^Department of General Affairs, Shenzhen Baoan Women’s and Children’s Hospital, Shenzhen, Guangdong, China; ^2^Department of Infectious Diseases, Nanfang Hospital, Southern Medical University, Guangzhou, China; ^3^Department of Spleen and Gastroenterology, Dongxihu District Hospital of Traditional Chinese Medicine, Wuhan, Hubei, China; ^4^School of Traditional Chinese Medicine, Hubei University of Chinese Medicine, Wuhan, Hubei, China; ^5^Department of Spleen and Gastroenterology, Hubei Provincial Hospital of Traditional Chinese Medicine, Affiliated Hospital of Hubei University of Chinese Medicine, Wuhan, Hubei, China; ^6^Hubei Shizhen Laboratory, Wuhan, Hubei, China

**Keywords:** colorectal adenomas, cross-sectional study, lifestyle, mental health, Wuhan

## Abstract

**Background:**

Colorectal adenomas, which are precancerous lesions that can develop into colorectal cancer, present a significant challenge due to the lack of comprehensive early screening and clear identification of risk factors.

**Objectives:**

We conduct a double-blind, prospective cross-sectional analysis to examine the relationship between lifestyle, mental health, and colorectal adenomas.

**Methods:**

Between June 2023 and July 2024, we surveyed 246 participants at Hubei Provincial Hospital of Traditional Chinese Medicine in Wuhan using a self-administered online questionnaire.

**Results:**

The majority of participants were over the age of 50 (49.6%), married or living with a partner (87.08%), and employed as office workers or technicians (44.3%). Among the total population, 435 individuals (53.5%) were diagnosed with colorectal adenomas. A significant positive association was observed between being a manager (OR = 2.340; 95% CI = 1.043–5.248) and the presence of colorectal adenomas, as well as having a BMI over 28 (OR = 6.000; 95% CI = 1.501–23.991). After adjusting for professional role and BMI, no significant associations were found between scores on the HADS-D (AOR = 1.031; 95% CI = 0.967–1.099) or PSS-10 (AOR = 0.971; 95% CI = 0.923–1.022) scales and colorectal adenomas. However, higher scores on the AUDIT (AOR = 1.001–1.144), CDS-12 (AOR = 1.028; 95% CI = 1.003–1.054), PSQI (AOR = 1.079; 95% CI = 1.003–1.161), and HADS-A (AOR = 1.156; 95% CI = 1.059–1.262) scales were significantly associated with an increased likelihood of colorectal adenomas.

**Conclusion:**

The study highlights the significance of addressing alcohol consumption, smoking, sleep quality, and anxiety to reduce the risk of colorectal adenomas. Targeted mental health interventions may play a crucial role in alleviating this health burden and enhancing overall population health.

## 1 Introduction

Colorectal cancer (CRC) is a significant global health challenge, ranking as the third most common cancer (1) and the second leading cause of cancer-related deaths worldwide (2). In China, the burden of CRC is particularly high, with the country accounting for 29% of all newly diagnosed cases globally, totaling 555,477 cases (2). Colorectal adenomas, which are the primary precursors of CRC, represent approximately 85–90% of sporadic CRC cases and pose a critical public health concern due to their potential to progress into cancer (3). Screening efforts have identified adenomas in up to 50% of asymptomatic individuals undergoing colonoscopy or CT colonography, highlighting the widespread nature of this condition (4). Of these adenomas, 3.4%–7.6% are classified as advanced, and 0.2%–0.6% are malignant (4). Screening tests, including surveillance colonoscopy, circulating plasma microRNAs, and a novel fecal *Lachnoclostridium* marker, have demonstrated potential in detecting colorectal adenomas (5–8) and early-stage cancer, contributing to reduced mortality rates (9). The increasing incidence of colorectal adenomas in China (10) underscores the need for enhanced screening and the identification of risk factors to mitigate this growing public health issue.

Recent research has underscored the significant impact of lifestyle factors on disease prevention and management (11). Increasing evidence suggests that lifestyle factors, including alcohol consumption (12–14), smoking (13, 15), and sleep quality (16, 17), are associated with an elevated risk of colorectal cancer. Globally, smoking and alcohol use were identified as the leading contributors to CRC disability-adjusted life years in 2019 (18). Additionally, circadian rhythm disruption plays a key role in tumorigenesis (19), and poor sleep quality has been associated with adverse health outcomes, including cardiovascular disease (20), obesity (21), and diabetes (22). Recent studies have also pointed out the significant positive relationship between sleep disturbances and colorectal adenomas (23, 24). These findings suggest that unhealthy lifestyle factors increase the risk of CRC. However, limited research in China has investigated the relationship between lifestyle factors and colorectal adenomas, which are precursors to CRC, particularly for early screening.

In addition to established risk factors like age (25) and family history (26), emerging research suggests that psychological factors may significantly influence the development and progression of colorectal adenomas (17, 27–33). There is a growing concern on the relationship between anxiety (31), depression (32), stress (33), and colorectal cancer. Chronic psychological distress can lead to immune system dysregulation (34) and increased systemic inflammation (35), both of which have been implicated in colorectal cancer pathogenesis (36, 37). Although psychological factors can increase the risk of CRC, few studies in China have specifically examined their association with colorectal adenomas.

**FIGURE 1:**
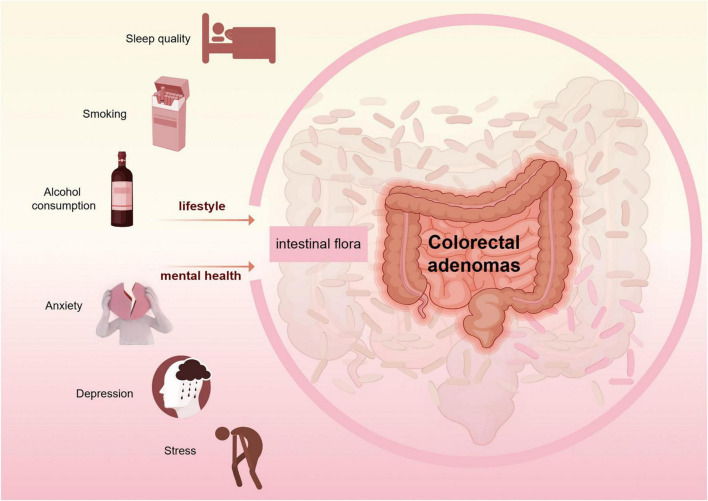
Schematic overview of the study design.

To address these gaps, we conducted a prospective cross-sectional survey in Wuhan, China, to examine the association between colorectal adenomas and demographic, lifestyle, and mental health factors among adults (Figure 1). We initially performed univariate logistic regression analysis and included variables with significant differences (*p* < 0.05) in a multivariate model for further evaluation. Through the use of multiple logistic regression models, our study aims to deepen understanding of the disease’s etiology and identify potential targets for intervention and prevention.

## 2 Materials and methods

### 2.1 Study design

Between June 2023 and July 2024, a cross-sectional survey was conducted among adults in Wuhan.

### 2.2 Sample size

Our sample size (*N*) was determined utilizing the formula: $N=\frac{Z^{2}\,p\,\left(1-p\right)}{d^{2}}$. Considering a desired confidence level of 95% (*Z* -score: 1.96), an estimated population proportion of 0.2 (*p*), and a desired margin of error of 0.05 (*d*), the minimum sample size (*N*) required is calculated to be 246 participants. Out of the selected 320 participants, 38 declined to participate in the study, and 36 withdrew from the study. The remaining 246 participants completed all self-administered online questionnaires that took approximately 20 min. The survey achieved a response rate of 76.9%.

### 2.3 Participants and data collection

Participants in this study were adults aged eighteen and over who underwent lower gastrointestinal endoscopy at Hubei Provincial Hospital of Traditional Chinese Medicine, including both inpatients and outpatients. The research team used a volunteer sampling approach to recruit participants. A questionnaire, developed by a panel of four gastroenterology clinicians, two epidemiologists, and one psychologist, was used to gather data. This questionnaire was created using Wenjuanxing and was initially evaluated with fifteen randomly selected participants. Based on their feedback, the panel made necessary adjustments to finalize the questionnaire. The questionnaire is available in the Supplementary material.

Before undergoing colonoscopy, all eligible participants completed the questionnaire without any knowledge of the microscopic findings and pathological results. Participants were invited to a dedicated consulting room where well-trained workers provided detailed information about the study and confirmed their eligibility. Participants were assured of their anonymity and informed of their right to withdraw from the study at any time without any consequences. Written informed consent was obtained from all participants prior to their inclusion in the study. Upon completing the survey, participants received a ¥20 (US$2.81) cash coupon as compensation for their time. There was no missing data in this study. All data and codes used in this study were available at https://github.com/BennyZhu2025/Cross_sectional_studies.git.

### 2.4 Ethics approval

The study received ethical approval from the Ethics Committee of Hubei Provincial Hospital of Traditional Chinese Medicine (Approval Number: HBZY2024-C29-02).

### 2.5 Measurements

#### 2.5.1 Background characteristics of the participants

Participants provided sociodemographic details encompassing age, gender, marital status, highest educational attainment, monthly income, occupation, and BMI.

#### 2.5.2 Dependent variables

Participants diagnosed with colorectal adenomas had initially been found to have polyps during their first lower gastrointestinal endoscopy, with postoperative pathology confirming the presence of adenomas. In contrast, both the endoscopic evaluations and postoperative pathology of patients without colorectal adenomas showed normal results. The diagnosis of colorectal adenomas was confirmed through pathological examination, considered the gold standard, while polyps were identified through endoscopic evaluation.

#### 2.5.3 Lifestyle variables

The Alcohol Use Disorder Identification and Testing Instrument, commonly referred to as AUDIT (38), contains 10 items designed to measure various aspects of alcohol consumption such as frequency and quantity as well as episodes of binge drinking (three items), symptoms associated with dependence (three), as well as its damaging social and health repercussions associated with its use (four). The overall Cronbach’s α (39) of AUDIT was 0.864 and the McDonald’s omega (40) was 0.897.

The Cigarette Dependence Scale (CDS-12) (41) is a meticulously designed 12-item questionnaire intended to assess specific dimensions of dependence. Notably, it examines indicators encompassing smoking compulsion, withdrawal symptoms, loss of control over time or activities that one used to enjoy and the persistence in smoking even when there is potential harm involved. Furthermore, CDS-12 includes items which capture self-perceptions of addiction or smoking rates with responses on a five-point Likert scale for each question. Cronbach’s alpha coefficients (39) were 0.983 for CDS-12 and the McDonald’s omega (40) was 0.985.

The Pittsburgh Sleep Quality Index (PSQI) (42), developed in 1989, is a validated questionnaire employed to assess sleep-related difficulties. The PSQI is administered as a self-report questionnaire that requires individuals to answer nineteen questions regarding their sleep patterns during the last month, plus five optional ones from cohabitants who share rooms or beds with them. This questionnaire aims to provide comprehensive insights into various aspects of sleep quality. Cronbach’s alpha coefficients (39) were 0.763 for PSQI and the McDonald’s omega (40) for this scale was 0.772.

#### 2.5.4 Mental health variables

The Hospital Anxiety and Depression Scale (HADS) (43) is a validated instrument comprising 14 items, specifically developed to evaluate symptoms of anxiety and depression among clinical patients. Notably, the scale aims to minimize the influence of physical illness on the overall score. The items pertaining to depression primarily target anhedonia symptoms commonly associated with depression. Participants rate each item on a 4-point severity scale, allowing for a comprehensive assessment of symptom severity. The overall Cronbach’s α (39) of HADS was 0.842 and the McDonald’s omega (40) for this scale was 0.848.

The Perceived Stress Scale-10 (PSS-10), originally developed by Cohen et al. (44), is an widely utilized 10-item questionnaire used to assess stress levels among individuals aged 12 years or over - this measure includes both children and adults alike. Response options are recorded using a five-point Likert scale from 0 to 4, where 4 corresponds with never; three with rarely; 2 with sometimes; 1 with frequently and 0 with always. Additionally, items 4, 5, 7, and 8 are positively scored. A total score of 13 indicates a normal stress level, while scores of 20 or higher denote elevated stress levels warranting therapeutic intervention (45). The Cronbach’s α (39) and McDonald’s omega (40) were 0.749 and 0.760, respectively.

### 2.6 Statistical analysis

The figure illustrating the schematic overview of the study design was created using Figdraw^1^. Statistical analyses were conducted using R (version 4.4.2) and Python (version 3.12.3) in the Microsoft Visual Studio Code environment. Categorical variables were encoded into numeric types using Scikit-learn’s LabelEncoder. Categorical variables were analyzed at each level using descriptive statistics, including frequencies and percentages. For continuous variables, means and standard deviations were reported. Univariate logistic regression was performed for all variables, followed by stepwise multivariate logistic regression for variables with significant differences in the univariate analysis. Multicollinearity among independent variables in each logistic regression model was assessed using the variance inflation factor (46). The linearity of the logit was assessed using the Box-Tidwell Test. Outliers were identified using Casewise Diagnostics, applying a 3-standard deviation default threshold. The adequacy of the multivariable models was evaluated using the Hosmer and Lemeshow goodness-of-fit tests (47). Overdispersion was examined using studentized permutations. A two-sided *p* < 0.05 was considered statistically significant. The Python environment was equipped with some packages, including pandas (version 2.1.4), numpy (version 1.24.3), scikit-learn (version 1.3.0), statsmodels (version 0.14.2) and scipy (version 1.11.4). Similarly, the R environment included necessary packages such as MASS (version 7.3-61), ltm (version 1.2-0), dplyr (version 1.1.4), tableone (version 0.13.2), openxlsx (version 4.2.7.1), and broom (version 1.0.7).

## 3 Results

### 3.1 Characteristics of participants

The characteristics of the participants are summarized in Table 1. Half of the participants were over 50 years old (49.6%), and the majority were either married or cohabiting (87.8%). Office workers and technicians made up the largest professional group (44.3%), and most participants had a BMI between 18 and 24 (54.9%). Additionally, 31.7% of the participants held a university degree or higher. Female participants represented 54.9% of the total sample, while 34.6% had a monthly income between 700 and 1,400. The study found that 107 participants (43.5%) were diagnosed with colorectal adenomas, while 139 (56.5%) were not.

**TABLE 1:** Characteristics of participants (*N* = 246).

	*n*	%
**Age (years)**		
18–30	23	9.3
31–40	47	19.1
41–50	54	22.0
> 50	122	49.6
**Gender**		
Male	111	45.1
Female	135	54.9
**Marital status**		
Single	21	8.5
Married or cohabited	216	87.8
Divorced, separated, or widowed	9	3.7
**Education level**		
Junior high or lower	66	26.8
Senior high or equivalent	46	18.7
College	56	22.8
University and above	78	31.7
**Monthly personal income ($)**		
< 420	50	20.3
420–700	72	29.2
700–1,400	85	34.6
> 1,400	39	15.9
**Professional role**		
Unemployed	40	16.3
Frontline worker	33	13.4
Office worker/technician	109	44.3
Manager	64	26.0
**Body Mass Index (BMI)**		
< 18	16	6.5
18–24	135	54.9
24–28	68	27.6
> 28	27	11.0
**Colorectal adenoma**		
Yes	107	43.5
No	139	56.5

### 3.2 Scale scores of lifestyle-level and mental health-level variables

**FIGURE 2:**
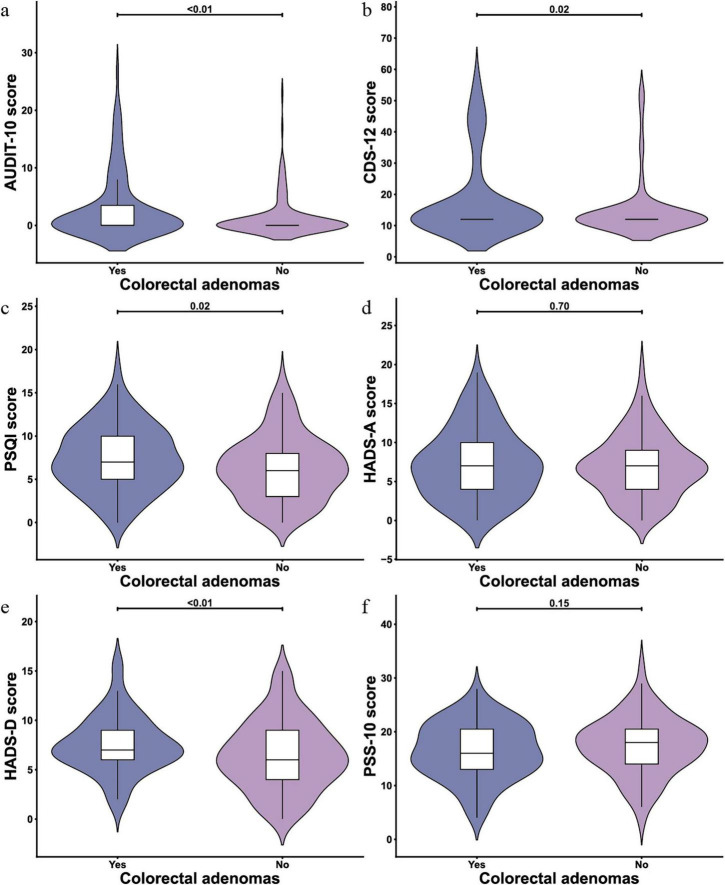
Violin plots of scale scores for lifestyle and mental health variables (*N* = 246). **(a)** AUDIT-10 scale; **(b)** Cigarette Dependence Scale (CDS)-12 scale; **(c)** Pittsburgh Sleep Quality Index (PSQI) scale; **(d)** Hospital Anxiety and Depression Scale (HADS)-A scale; **(e)** HADS-D scale; **(f)** Perceived Stress Scale-10 (PSS-10) scale.

The lifestyle-related scales showed an average score of 2.10 (SD 4.58) on the AUDIT scale, 16.42 (SD 11.00) on the CDS-12 scale, and 6.78 (SD 3.77) on the PSQI scale. Regarding mental health, the HADS-A subscale had a mean score of 7.09 (SD 4.21), the HADS-D sub-scale averaged 6.94 (SD 3.31), and the PSS-10 scale recorded a mean score of 16.15 (SD 3.94). These results are detailed in Figure 2.

### 3.3 Background characteristics associated with colorectal adenomas

Table 2 shows that there are significant positive associations between being a manager (OR = 2.340; 95% CI = 1.043–5.248) and colorectal adenomas, as well as having a BMI over 28 (OR = 6.000; 95% CI = 1.501–23.991) and colorectal adenomas. However, no significant correlations were found between colorectal adenomas and factors such as age, gender, marital status, education level, or monthly income.

**TABLE 2:** Associations between demographic factors and colorectal adenomas (*N* = 246).

	OR (95% CI)	*P*-value
**Age (years)**		
18–30	Reference	
31–40	2.288 (0.766, 6.834)	0.138
41–50	2.443 (0.835, 7.146)	0.103
> 50	2.326 (0.858, 6.301)	0.097
**Gender**		
Male	Reference	
Female	0.730 (0.439, 1.212)	0.223
**Marital status**		
Single	Reference	
Married or cohabited	2.658 (0.940, 7.514)	0.065
Divorced, separated, or widowed	2.560 (0.489, 13.389)	0.265
**Education level**		
Junior high or lower	Reference	
Senior high or equivalent	1.952 (0.909, 4.192)	0.086
College	1.640 (0.796, 3.377)	0.179
University and above	0.971 (0.493, 1.910)	0.931
**Monthly personal income ($)**		
< 420	Reference	
420–700	1.419 (0.683, 2.946)	0.384
700–1,400	0.861 (0.420, 1.765)	0.683
> 1,400	1.750 (0.751, 4.080)	0.195
**Professional role**		
Unemployed	Reference	
Frontline worker	1.105 (0.433, 2.819)	0.834
Office worker/technician	0.803 (0.381, 1.691)	0.564
Manager	2.340 (1.043, 5.248)	0.039
**Body Mass Index (BMI)**		
< 18	Reference	
18–24	1.939 (0.594, 6.330)	0.273
24–28	2.667 (0.781, 9.102)	0.117
> 28	6.000 (1.501, 23.991)	0.011

### 3.4 Lifestyle-level and mental health-level scales associated with colorectal adenomas

Before adjusting for demographic variables, significant associations with colorectal adenomas were identified across various measurement scales. For lifestyle factors, AUDIT showed a significant relationship (OR = 1.097, 95% CI: 1.030–1.168), as did CDS-12 (OR = 1.030, 95% CI: 1.005–1.055) and PSQI (OR = 1.083, 95% CI: 1.012–1.160). Among mental health assessments, HADS-A was notably correlated with colorectal adenomas (OR = 1.108, 95% CI: 1.024–1.199), while HADS-D did not show a significant connection (OR = 1.017, 95% CI: 0.958–1.080). Additionally, no significant association was observed between PSS-10 (OR = 0.966, 95% CI: 0.921–1.013) and colorectal adenomas.

After controlling for professional role and BMI, higher AUDIT scores remained significantly associated with colorectal adenomas (AOR = 1.070; 95% CI: 1.001–1.144). Significant associations were also found between CDS-12 scores (AOR = 1.028; 95% CI: 1.003–1.054) and PSQI scores (AOR = 1.079; 95% CI = 1.003–1.161) with colorectal adenomas. The HADS-A subscale continued to show a significant association (AOR = 1.156; 95% CI: 1.059–1.262), while the HADS-D subscale did not (AOR = 1.031; 95% CI: 0.967–1.099). Furthermore, no significant association was found between PSS-10 scores (AOR = 0.971; 95% CI: 0.923–1.022) and colorectal adenomas. These findings are detailed in Table 3.

**TABLE 3:** Lifestyle-level and mental health-level scales associated with colorectal adenomas (*N* = 246).

	OR (95% CI)	*P*-value	AOR (95% CI)	*P*-value
**Lifestyle-level scales**				
AUDIT	1.097 (1.030, 1.168)	0.004	1.070 (1.001, 1.144)	0.047
CDS-12	1.030 (1.005, 1.055)	0.017	1.028 (1.003, 1.054)	0.029
PSQI	1.083 (1.012, 1.160)	0.022	1.079 (1.003, 1.161)	0.042
**Mental health-level scales**				
HADS-A	1.108 (1.024, 1.199)	0.011	1.156 (1.059, 1.262)	0.001
HADS-D	1.017 (0.958, 1.080)	0.573	1.031 (0.967, 1.099)	0.349
PSS-10	0.966 (0.921, 1.013)	0.151	0.971 (0.923, 1.022)	0.260

## 4 Discussion

By integrating pathological examinations, endoscopic evaluations, and validated questionnaire data, our study provides a comprehensive understanding of the factors contributing to colorectal adenomas. This multi-faceted approach enhances the reliability of our findings by allowing for cross-validation and reducing biases inherent in single-method studies. The use of validated scales to assess lifestyle and mental health factors further strengthens our results, enabling the identification of significant associations with colorectal adenoma risk, such as alcohol consumption, smoking, sleep quality, and anxiety. These findings underscore the importance of incorporating both lifestyle and mental health considerations into routine screening and prevention strategies, offering a robust foundation for future research and the development of more effective interventions to reduce the burden of colorectal cancer.

The survey was conducted in Wuhan to investigate the prevalence and risk factors associated with colorectal adenomas among adults. The study population demonstrated a colorectal adenoma prevalence rate of 43.5% in Wuhan, China. Interestingly, consistent with previous research work (48, 49), no significant associations were observed between age, marital status, gender, education level, and monthly personal income, and colorectal adenomas. However, study findings revealed the professional roles played by managers as an influential modifiable risk factor for colon adenomas development. Furthermore, our investigation revealed a statistically significant association between a BMI exceeding 28 and colorectal adenomas, suggesting potential variations in the exposure to risk factors.

The present study has revealed significant positive associations between lifestyle factors, alcohol consumption, smoking, and colorectal adenomas. While previous studies have focused on lifestyle factors such as alcohol abuse and smoking (50–52), there remains limited research exploring the relationship between sleep quality and colorectal adenomas. lifestyle factors may disrupt various biological processes, including immune function, DNA repair mechanisms, and gut microbiota composition, all of which could contribute to the development and progression of colorectal adenomas (53). Consequently, further comprehensive investigations, including basic research and well-designed cohort studies, are indispensable for a more thorough understanding of this subject matter.

Our study makes a groundbreaking investigation into the influence of mental health on the screening risk factors associated with colorectal adenomas. While prior research conducted by Aceto et al. has shed some light on the potential microenvironmental alterations resulting from poor mental health, which may promote colorectal adenomas (54), the precise relationship between mental health and colorectal adenomas remains elusive. In our study, we provide compelling and interesting evidence that underscores the significant role of anxiety in the likelihood of developing colonic adenomatous polyps. Notably, emerging research indicates that anxiety exerts distinct effects on the patient’s immune system, metabolism, and physiological functions (55). Therefore, placing paramount importance on fostering a positive attitude becomes crucial in reducing the incidence of colorectal adenomatous polyps. Furthermore, disseminating the concept that an optimistic mood can enhance intestinal function holds immense promise as a potentially efficacious strategy. By elucidating the intricate association between mental health and colorectal adenomas, our findings contribute to a deeper comprehension of the multifaceted factors underpinning the etiology of this condition.

Our work offers a meaningful contribution toward reducing the medical burden associated with colorectal adenomas and minimizing the need for lower gastrointestinal endoscopy. By recognizing the association between mental health and colorectal adenomas, healthcare providers can adopt more comprehensive and tailored approaches to prevention and management. However, further research is necessary to elucidate the underlying mechanisms through which mental health influences colorectal adenomas. This would facilitate the development of targeted interventions that promote both mental well-being and gastrointestinal health. Furthermore, we advocate for the integration of lifestyle and mental health considerations into colorectal adenoma screening protocols, allowing for more comprehensive and tailored approaches to prevention and management. Through these efforts, we can address the multifaceted factors contributing to colorectal adenomas, reducing the burden of this condition and improving population health outcomes.

Our research establishes a scientific basis for enhancing colorectal adenoma screening, prevention strategies, and targeted interventions by incorporating lifestyle and mental health considerations into clinical practice. Recent studies support the integration of these factors, indicating that lifestyle choices such as diet and physical activity significantly impact adenoma risk, thereby suggesting that personalized interventions could improve screening outcomes (56). Additionally, mental health factors, including stress and anxiety, have been linked to health behaviors that influence colorectal cancer risk, underscoring the importance of a holistic approach in clinical settings (57, 58). Community-based interventions that address both lifestyle and mental health components have also demonstrated potential in increasing screening rates and patient engagement (59). Despite these advances, challenges remain in effectively implementing these strategies across diverse populations, highlighting the need for further research to optimize and tailor interventions (60). Therefore, our research contributes to the ongoing effort to refine and improve colorectal adenoma prevention and care.

This study has some limitations. Firstly, the study did not fully account for confounders like comorbidities, diet, and genetics. Conditions such as diabetes or inflammatory bowel disease, along with dietary factors like fiber intake, could independently affect both psychological states and colorectal adenoma development, potentially skewing results. Similarly, genetic predispositions could influence both psychological resilience and adenoma growth. Future research should gather detailed clinical, dietary, and genetic data, using standardized tools and advanced statistical methods to adjust for these factors. Collaboration with multidisciplinary teams would help clarify whether observed relationships are causal or arise from shared underlying mechanisms. Secondly, our research did not collect data from participants who declined to take part. Thirdly, no statistical analyses were performed regarding specific polyp locations, sizes or postoperative pathological classification for patients diagnosed with colorectal polyps. Fourthly, even though the study was anonymous, patients’ self-report might cause evaluation bias, which can be reduced by computer assistants. Moreover, we confined our sample to patients undergoing their first colonoscopy. As the perfection of examination and follow-up treatment intervention would have led to changes in patients’ mood and lifestyle, causing errors. Ideally, our data analysis should have focused on stratifying patients according to whether they had previously undergone colonoscopy and completed subsequent treatment after examination. Furthermore, since our study used cross-sectional design with no longitudinal data collection we cannot draw definitive conclusions regarding causal links among depression, individual press, sleep quality and colorectal adenomas.

## 5 Conclusion

This study identified significant correlations between alcohol consumption, smoking, sleep quality, anxiety, and the occurrence of colorectal adenomas among adults in China. These findings highlight the importance of targeted mental health interventions, which could help reduce the incidence of colorectal adenomas and improve overall public health outcomes.

## Data Availability

The datasets presented in this study can be found in online repositories. The names of the repository/repositories and accession number(s) can be found in the article/Supplementary material.

## Footnotes

1
https://www.figdraw.com
