# Supplementary material for: Impact of lifestyle and mental health on colorectal adenomas in China: a prospective cross-sectional survey
Source: Front Med (Lausanne). 2025 Mar 3;12:1475987. doi: 10.3389/fmed.2025.1475987 (PMC11911329; doi:10.3389/fmed.2025.1475987)
Supplement: Supplementary file 1 [file Supplementary_file_1.pdf]

Translation for reference:

Questionnaire for Colonoscopy

Part Ⅰ,Basic Information((a total of 16 questions)

1. Gender

✧ Female

✧ Male

2. Age

✧ ___________

3. Hospitalization Number (outpatients please fill in "0")

✧ ___________

4. Height(cm)

✧ ___________

5. Body weight (kg)

✧ ___________

6. Contact phone number

✧ ___________

7. Native place (province, city)

✧ ___________

8. Current place of residence

✧ Wuhan

✧ Other counties and cities in Hubei Province

✧ Other province

9. Marital status

✧ Unmarried

✧ Married

✧ Divorced

✧ Widowed

10. Educational level

✧ Primary school and below

✧ Junior high school / Secondary technical school

✧ Senior high school / Higher vocational college

✧ Junior college

✧ Bachelor's degree and above

11. Occupation

✧ Unemployed

✧ Manual laborers

✧ Clerks /Technical

✧ managerial staff

12. Monthly income

✧ Below 3,000
✧ 3,000 - 4,999
✧ 5,000 - 9,999
✧ Over 10,000

13. Rating of household residential noise (0 points indicates comfortable and noise-free; 10 points indicates extremely noisy and unbearable)

0 10

★ ★ ★ ★ ★ ★ ★ ★ ★ ★ ★

PartⅡ,Gastrointestinal symptoms (a total of 15 questions)

14. In the past week, have you been troubled by pain or discomfort in the upper abdomen or epigastrium?

✧ No discomfort
✧ Slight discomfort
✧ Mild discomfort
✧ Moderate discomfort
✧ Somewhat severe discomfort
✧ Severe discomfort
✧ Extremely severe discomfort

15. Have you been troubled by heartburn in the past week?

✧ No discomfort
✧ Slight discomfort
✧ Mild discomfort
✧ Moderate discomfort
✧ Slightly severe discomfort
✧ Severe discomfort
✧ Extremely severe discomfort

16. Have you been troubled by acid reflux in the past week?

✧ No discomfort
✧ Slight discomfort
✧ Mild discomfort
✧ Moderate discomfort
✧ Somewhat severe discomfort
✧ Severe discomfort
✧ Extremely severe discomfort

17. Have you been troubled by hungry stomach pain in the past week?

✧ No discomfort
✧ Slight discomfort
✧ Mild discomfort
✧ Moderate discomfort

- ✧ Somewhat severe discomfort
- ✧ Severe discomfort
- ✧ Extremely severe discomfort

18. Have you been bothered by nausea in the past week?

- ✧ No discomfort
- ✧ Slight discomfort
- ✧ Mild discomfort
- ✧ Moderate discomfort
- ✧ Slightly severe discomfort
- ✧ Severe discomfort
- ✧ Extremely severe discomfort

19. Have you been bothered by the gurgling sounds in your stomach in the past week?

- ✧ No discomfort
- ✧ Slight discomfort
- ✧ Mild discomfort
- ✧ Moderate discomfort
- ✧ Somewhat severe discomfort
- ✧ Severe discomfort
- ✧ Extremely severe discomfort

20. Have you felt bloated in the past week?

- ✧ No discomfort
- ✧ Slight discomfort
- ✧ Mild discomfort
- ✧ Moderate discomfort
- ✧ Slightly severe discomfort
- ✧ Severe discomfort
- ✧ Extremely severe discomfort

21. Have you been troubled by hiccups in the past week?

- ✧ No discomfort
- ✧ Slight discomfort
- ✧ Mild discomfort
- ✧ Moderate discomfort
- ✧ Somewhat severe discomfort
- ✧ Severe discomfort
- ✧ Extremely severe discomfort

22. Have you been bothered by flatulence or passing gas in the past week?

- ✧ No discomfort
- ✧ Slight discomfort
- ✧ Mild discomfort
- ✧ Moderate discomfort
- ✧ Somewhat severe discomfort
- ✧ Severe discomfort
- ✧ Extremely severe discomfort

23. Have you been troubled by constipation in the past week?

- ✧ No discomfort
- ✧ Slight discomfortMild discomfort
- ✧ Moderate discomfort
- ✧ Slightly severe discomfort
- ✧ Severe discomfort
- ✧ Extremely severe discomfort

24. Have you been troubled by diarrhea in the past week?

- ✧ No discomfort
- ✧ Slight discomfort
- ✧ Mild discomfort
- ✧ Moderate discomfort
- ✧ Somewhat severe discomfort
- ✧ Severe discomfort
- ✧ Extremely severe discomfort

25. In the past week, have you been troubled by loose stools that alternate between dry and thin?

- ✧ No discomfort
- ✧ Slight discomfort
- ✧ Mild discomfort
- ✧ Moderate discomfort
- ✧ Mildly severe discomfort
- ✧ Severe discomfort
- ✧ Extremely severe discomfort

26. In the past week, have you been troubled by stools that are sometimes dry and hard and sometimes loose?

- ✧ No discomfort
- ✧ Slight discomfort
- ✧ Mild discomfort
- ✧ Moderate discomfort
- ✧ Slightly severe discomfort
- ✧ Severe discomfort
- ✧ Extremely severe discomfort

27. Have you been bothered by a sense of urgency to defecate in the past week?

- ✧ No discomfort
- ✧ Slight discomfort
- ✧ Mild discomfort
- ✧ Moderate discomfort
- ✧ Somewhat severe discomfort
- ✧ Severe discomfort
- ✧ Extremely severe discomfort

28. Have you been bothered by a feeling of incomplete defecation in the past week?

- ✧ No discomfort
- ✧ Slight discomfort
- ✧ Mild discomfort
- ✧ Moderate discomfort
- ✧ Slightly severe discomfort
- ✧ Severe discomfort
- ✧ Extremely severe discomfort

Part Ⅲ, Drinking Situation (10 questions in total)

29. How often did you drink alcohol in the past year?

- ✧ Never drink alcohol
- ✧ Once a month or once every few months
- ✧ 2 - 4 times a month
- ✧ 2 - 3 times a week
- ✧ 4 times a week or more

30. On the days when you drink, how much Chinese liquor do you usually drink? (1 tael = 50ml)

- ✧ Don't drink Chinese liquor
- ✧ 1 - 2 taels
- ✧ 3 - 4 taels
- ✧ 5 - 6 taels
- ✧ 7 - 8 taels
- ✧ Over 9 taels

31. On the day when you drink, how many cans of beer do you usually drink? (One can is about 330ml)

- ✧ Don't drink beer
- ✧ 1 - 2 cans
- ✧ 3 - 4 cans
- ✧ 5 - 6 cans
- ✧ 7 - 9 cans
- ✧ More than 10 cans

32. On the days when you drink, how much red wine do you usually drink? (1 glass = 150ml)

- ✧ Don't drink red wine
- ✧ 1-2 glasses
- ✧ 3 - 4 glasses
- ✧ 5 - 6 glasses
- ✧ 7 - 9glasses
- ✧ Over 10 glasses

33. How often do you have an occasion where you drink more than: 6 cans of beer, or 6 taels of Chinese liquor, or 6 glasses of red wine?

- ✧ Never
- ✧ Once every two months or several months

- ✧ Once a month
- ✧ Once a week
- ✧ Once a day, or nearly once a day

34. In the past year, how often did the following situation occur: Finding that you couldn't stop drinking once you started?

- ✧ Never
- ✧ Once every two months or several months
- ✧ Once a month
- ✧ Once a week
- ✧ Once a day, or nearly once a day

35. In the past year, how often did the following situations occur: Missed planned activities because of drinking

- ✧ Never
- ✧ Once every two months or several months
- ✧ Once a month
- ✧ Once a week
- ✧ Once a day, or nearly once a day

36. In the past year, how often did the following situations occur: After heavy drinking, one must have a morning drink (early morning alcohol) to regain their spirits.

- ✧ Never
- ✧ Once every two months or several months
- ✧ Once a month
- ✧ Once a week
- ✧ Once a day, or nearly once a day

37. In the past year, how often did the following situation occur: feeling guilty or self - reproachful after drinking alcohol?

- ✧ Never
- ✧ Once every two months or several months
- ✧ Once a month
- ✧ Once a week
- ✧ Once a day, or nearly once a day

38. In the past year, how often did the following situation occur: Forgetting what happened the previous night after drinking?

- ✧ Never
- ✧ Once every two months or several months
- ✧ Once a month
- ✧ Once a week
- ✧ Once a day, or nearly once a day

39. Do you have the following situations:Have you ever hurt yourself or others because of drinking?

- ✧ No
- ✧ Yes, but not in the past year
- ✧ Yes, in the past year

40. Do you have the following situations:Have your relatives, friends, doctors or other health - care workers ever shown concern about your drinking problem or advised you to drink less?

- ✧ No
- ✧ Yes, but not in the past year
- ✧ Yes, in the past year

Part IV,Smoking Status (a total of 13 questions)

41. Do you smoke?

- ✧ Non - smoker
- ✧ Smoker

42. Please rate your level of nicotine addiction (0 means no addiction, 100 means extremely addicted)

0 ○ ○ ○ ○ ○ ○ ○ ○ ○ ○ ○ 100

43. How many cigarettes do you smoke on average per day?

- ✧ 0 - 5 cigarettes
- ✧ 6 – 10 cigarettes
- ✧ 11 – 20 cigarettes
- ✧ 21 – 29 cigarettes○ More than 30 cigarettes

44. How long after you get up do you smoke your first cigarette?

- ✧ 0 - 5 minutes
- ✧ 6 - 15 minutes
- ✧ 16 - 30 minutes
- ✧ 31 - 60 minutes
- ✧ After 60 minutes

45. How difficult is it for you to quit smoking?

- ✧ Completely impossible
- ✧ Very difficult
- ✧ Somewhat difficult
- ✧ Relatively easy
- ✧ Very easy

46. Do you agree with the following statement: After not smoking for a few hours, I will have an urgent and irresistible urge to smoke

- ✧ Completely disagree
- ✧ Somewhat disagree
- ✧ Neither agree nor disagree
- ✧ Slightly agree
- ✧ Completely agree

47. Do you agree with the following statement:I feel stressed at the thought of not having cigarettes.

- ✧ Completely disagree
- ✧ Slightly disagree
- ✧ Neither agree nor oppose.
- ✧ Slightly agree
- ✧ Completely agree

48. Do you agree with the following statement: Before going out, I always make sure to carry cigarettes with me at all times.

- ✧ Completely disagree
- ✧ Somewhat disagree
- ✧ Neither agree nor disagree
- ✧ Slightly agree
- ✧ Fully agree

49. Do you agree with the following statement: I've become a captive of cigarettes?

- ✧ Strongly disagree
- ✧ Somewhat disagree
- ✧ Neither agree nor disagree
- ✧ Slightly agree
- ✧ Strongly agree

50. Do you agree with the following statement: I feel I smoke too much?

- ✧ Strongly disagree
- ✧ Somewhat disagree
- ✧ Neither agree nor disagree
- ✧ Slightly agree
- ✧ Strongly agree

51. Do you agree with the following statement:Sometimes I'll put aside everything and go out to buy cigarettes?

- ✧ Totally disagree
- ✧ Somewhat disagree
- ✧ Neither agree nor oppose
- ✧ Slightly agree
- ✧ Fully agree

52. Do you agree with the following statement:I've been smoking all the time?

- ✧ Fully disagree
- ✧ Somewhat disagree
- ✧ Neither agree nor oppose
- ✧ Slightly agree
- ✧ Completely agree

53. Do you agree with the following statement:Even though it will harm my physical health, I will still choose to smoke

- ✧ Completely disagree

- ✧ A little bit disagree
- ✧ Neither agree nor oppose
- ✧ Slightly agree
- ✧ Fully agree

Part Ⅴ, Personal Stress Situation (10 questions in total)

54. In the past month, how often did the following event occur: You were upset by unexpected things

- ✧ Not at all
- ✧ Almost never
- ✧ Sometimes
- ✧ Often
- ✧ Most of the time

55. In the past month, how often did the following event occur: You felt unable to control important things in your life

- ✧ Not at all
- ✧ Almost never
- ✧ Sometimes
- ✧ Often
- ✧ Most of the time

56. In the past month, how often did the following event occur: You felt nervous or stressed

- ✧ Not at all
- ✧ Almost never
- ✧ Sometimes
- ✧ Often
- ✧ Most of the time

57. In the past month, how often did the following event occur: You felt confident in your ability to handle personal problems

- ✧ Not at all
- ✧ Almost never
- ✧ Sometimes
- ✧ Often
- ✧ Most of the time

58. In the past month, how often did the following event occur: Feeling that things are developing in the direction you planned

- ✧ Not at all
- ✧ Almost never
- ✧ Sometimes
- ✧ Often
- ✧ Most of the time

59. In the past month, how often did the following event occur: Feeling unable to cope with what you have to do

- ✧ Not at all
- ✧ Almost never
- ✧ Sometimes
- ✧ Often
- ✧ Most of the time

60. In the past month, how often did the following event occur: You felt that you could control the annoying things in your life?

- ✧ Not at all
- ✧ Almost never
- ✧ Sometimes
- ✧ Often
- ✧ Most of the time

61. In the past month, how often did the following event occur: You felt that you were in control of everything

- ✧ Not at all
- ✧ Almost never
- ✧ Sometimes
- ✧ Often
- ✧ Most of the time

62. In the past month, how often did the following events occur: Feeling angry because something happened beyond your control

- ✧ Not at all
- ✧ Almost never
- ✧ Sometimes
- ✧ Often
- ✧ Most of the time

63. In the past month, how often did the following events occur: Feeling that difficulties piled up so much that you couldn't overcome them.

- ✧ Not at all
- ✧ Almost never
- ✧ Sometimes
- ✧ Often
- ✧ Most of the time

Part VI, Sleep Status (23 questions in total)

Your answers should represent your situation for most days and nights in the past month.

64. In the past month, what time did you usually go to bed at night?

- ✧ __________

65. In the past month, how many minutes did you usually take to fall asleep? (Please fill in the number of minutes)

- ✧ __________

66. . In the past month, what time did you usually get up in the morning?

✧ __________

67. In the past month, how many hours did you actually sleep at night?

✧ __________

68. In the past month, how often did you have poor sleep due to the following reason:Unable to fall asleep within 30 minutes

✧ Did not occur in the past month
✧ Once every two weeks or several weeks
✧ 1 - 2 times a week
✧ 3 times a week or more

69. In the past month, how often did you have poor sleep due to the following reasons:Waking up in the middle of the night or early morning

✧ Did not occur in the past month
✧ Once every two weeks or several weeks
✧ 1 - 2 times a week
✧ 3 times a week or more

70. In the past month, how often did you have poor sleep due to the following reason: having to get up to go to the toilet during sleep?

✧ Did not occur in the past month
✧ Once every two weeks or several weeks
✧ 1 - 2 times a week
✧ 3 times a week or more

71. In the past month, how often did you have poor sleep due to the following reasons:Difficulty breathing

✧ Did not occur in the past month
✧ Once every two weeks or several weeks
✧ 1 - 2 times a week
✧ 3 times a week or more

72. In the past month, how often did you have poor sleep due to the following reasons: Loud coughing or snoring

✧ Did not occur in the past month
✧ Once every two weeks or several weeks
✧ 1 - 2 times a week
✧ 3 times a week or more

73. In the past month, how often did you have poor sleep due to the following reasons:Feeling too cold

✧ Did not occur in the past month
✧ Once every two weeks or several weeks
✧ 1 - 2 times a week
✧ 3 times a week or more

74. In the past month, how often did you have poor sleep due to the following reasons: Feeling too hot

✧ Did not occur in the past month

✧ Once every two weeks or several weeks
✧ 1 - 2 times a week
✧ 3 times a week or more

75. In the past month, how often did you have poor sleep due to the following reasons:Nightmares
✧ Did not occur in the past month
✧ Once every two weeks or several weeks
✧ 1 - 2 times a week
✧ 3 times a week or more

76. In the past month, how often did you have poor sleep due to the following reasons: Self - perceived pain
✧ Did not occur in the past month
✧ Once every two weeks or several weeks
✧ 1 - 2 times a week
✧ 3 times a week or more

77. In the past month, have you had poor sleep due to "other reasons" apart from the issues mentioned above? If so, please describe the reasons.
✧ ______________________

78. In the past month, how often did you have poor sleep due to "other reasons"?
✧ It didn't occur in the past month.
✧ Once every two weeks or several weeks.
✧ One to two times a week.
✧ More than 3 times a week

79. How would you rate your overall sleep in the past month?
✧ Very good
✧ Just so - so
✧ Not very good
✧ Very poor

80. In the past month, how often did you take medicine to help you fall asleep?
✧ Did not occur in the past month
✧ Once every two weeks or several weeks
✧ One to two times a week
✧ Three times a week or more

81. In the past month, how often did you have difficulty staying awake while driving, eating, or participating in social activities?
✧ Did not occur in the past month
✧ Once every two weeks or several weeks
✧ One to two times a week
✧ Three times a week or more

82. In the past month, for you, how much of a problem was it to maintain enough enthusiasm to complete tasks?

- ✧ No problem at all
- ✧ There are only minor problems
- ✧ There are some problems
- ✧ There are major problems

83. Do you have a partner or a roommate?

- ✧ Do not have a partner or a roommate
- ✧ Have a partner or a roommate, but in a different room
- ✧ The partner is in the same room, but not in the same bed
- ✧ Have a partner and sleep in the same bed

84. Ask your partner/roommate how often you have thefollowing behaviors in the past month: Loud snoring

- ✧ Did not occur in the past month
- ✧ Once every two weeks or several weeks
- ✧ One to two times a week
- ✧ Three times a week or more

85. Ask your partner/roommate to tell you how often you had the following behavior in the past month: Long pauses between breaths while sleeping

- ✧ Did not occur in the past month
- ✧ Once every two weeks or several weeks
- ✧ One to two times a week
- ✧ Three times a week or more

86. Ask your partner/roommate to tell you how often you had the following behavior in the past month: Leg twitching or cramps while sleeping.

- ✧ Did not occur in the past month
- ✧ Once every two weeks or several weeks
- ✧ One to two times a week
- ✧ Three times a week or more

87. Ask your partner/roommate to tell you how often you had the following behaviors in the past month: Disorientation or confusion during sleep

- ✧ Did not occur in the past month
- ✧ Once every two weeks or several weeks
- ✧ One to two times a week
- ✧ Three times a week or more

Part VII, Anxiety Situation (14 questions in total)

Check the response that is closest to how you felt in the past week. Your first impression is the best answer.

88. Feeling nervous

- ✧ Most of the time
- ✧ Most of the time; Quite often

- ✧ Sometimes; Occasionally
- ✧ Not at all

89. Still enthusiastic about what one used to like

- ✧ Completely true
- ✧ Interested, but not that passionate
- ✧ Only a little interested
- ✧ Almost no interest

90. There is a premonition of fear, as if something terrible is about to happen

- ✧ Very sure, and it's very bad
- ✧ Yes, but things aren't too bad
- ✧ I have such a hunch, but I'm not worried.
- ✧ Not at all

91. I'm very optimistic and can see the interesting side of things.

- ✧ Always like this.
- ✧ Yes, but not as much as before.
- ✧ Nowhere near as good as before.
- ✧ Not at all

92. Anxious thoughts linger in the mind

- ✧ Most of the time
- ✧ Most of the time
- ✧ Sometimes
- ✧ Occasionally

93. I feel very happy

- ✧ Not at all
- ✧ Rarely
- ✧ Sometimes
- ✧ Most of the time

94. I can sit calmly and feel very relaxed

- ✧ Exactly so
- ✧ Often
- ✧ Occasionally
- ✧ Not at all

95. I feel like I've become duller.

- ✧ Basically, it's always like this.
- ✧ Most of the time
- ✧ Sometimes
- ✧ Not at all

96. Feeling anxious and restless, with a fluttering heart.

- ✧ Not at all
- ✧ Occasionally
- ✧ Often
- ✧ Most of the time

97. I no longer pay attention to my appearance.

- ✧ Completely true
- ✧ Rarely pay attention
- ✧ Still pay attention, but less than before
- ✧ I always pay attention to my appearance as usual.

98. I feel anxious when I have to keep doing things.

- ✧ That's really the case.
- ✧ Most of the time
- ✧ Not very often
- ✧ Not at all

99. I look forward to enjoying things.

- ✧ As eager as before.
- ✧ Relatively less than in the past.
- ✧ Far less than before.
- ✧ Not at all.

100. Suddenly, I have a feeling of panic.

- ✧ Most of the time
- ✧ Most of the time; Quite often
- ✧ Occasionally
- ✧ Not at all

101. I will enjoy reading a good book, listening to the radio, or watching a TV program.

- ✧ Frequently,
- ✧ sometimes,
- ✧ occasionally,
- ✧ rarely

肠镜问卷

该问卷主要研究疾病发病因素和疾病的关系，所有个人信息均会保密，仅供研究者使用。您如果同意参与该项研究，请继续作答以下问卷，请如实回答相关问题。如果不同意参与该项目研究，直接退出即可。

第一部分，基本信息（共16项问题）

*1. 性别

○ 男

○ 女

*2. 年龄

*3. 住院号（门诊患者请填“0”）

*4. 身高（cm）

*5. 体重（kg）

*6. 联系电话

*7. 籍贯（省、市）

*8. 现居住地

○ 武汉市

○ 湖北省其他县市

○ 其他省份

*9. 婚姻状态

○ 未婚
○ 已婚
○ 离异
○ 丧偶

*10. 学历水平

○ 小学及以下
○ 初中 / 中专
○ 高中 / 高专
○ 大专
○ 本科及以上

*11. 职业

○ 待业
○ 体力劳动者
○ 职员 / 技术人员
○ 管理人员

*12. 月收入

○ 3000以下
○ 3000–4999
○ 5000–9999
○ 10000以上

*13. 家庭居住噪音评分（0分表示舒适无噪音；10分表示非常嘈杂难以忍受）

0 ★ ★ ★ ★ ★ ★ ★ ★ ★ ★ ★ 10

第二部分，胃肠道症状（共15项问题）
这项调查包含了您的主观感受和近一周内的变化

*14. 在过去的一周里，您是否被上腹部、胃脘部疼痛或不适困扰？

○ 无不适
○ 稍微不适
○ 轻度不适
○ 中度不适
○ 稍严重不适

○ 严重不适

○ 极其严重不适

*15. 在过去的一周里，您是否被烧心困扰?

○ 无不适

○ 稍微不适

○ 轻度不适

○ 中度不适

○ 稍严重不适

○ 严重不适

○ 极其严重不适

*16. 在过去的一周里，您是否被反酸困扰?

○ 无不适

○ 稍微不适

○ 轻度不适

○ 中度不适

○ 稍严重不适

○ 严重不适

○ 极其严重不适

*17. 在过去的一周里，是否被胃部饥饿痛困扰?

○ 无不适

○ 稍微不适

○ 轻度不适

○ 中度不适

○ 稍严重不适

○ 严重不适

○ 极其严重不适

*18. 在过去的一周里，您是否被恶心欲吐感困扰?

○ 无不适

○ 稍微不适

○ 轻度不适

○ 中度不适

○ 稍严重不适

○ 严重不适

○ 极其严重不适

*19. 在过去的一周里，您是否被胃中的咕咕声困扰?

- 无不适
- 稍微不适
- 轻度不适
- 中度不适
- 稍严重不适
- 严重不适
- 极其严重不适

*20. 在过去的一周里，是否感觉胃胀?

- 无不适
- 稍微不适
- 轻度不适
- 中度不适
- 稍严重不适
- 严重不适
- 极其严重不适

*21. 在过去的一周里，您是否被打嗝困扰?

- 无不适
- 稍微不适
- 轻度不适
- 中度不适
- 稍严重不适
- 严重不适
- 极其严重不适

*22. 在过去的一周里，您是否被排气或放屁困扰?

- 无不适
- 稍微不适
- 轻度不适
- 中度不适
- 稍严重不适
- 严重不适
- 极其严重不适

*23. 在过去的一周里，您是否被便秘困扰?

- 无不适
- 稍微不适

◯ 轻度不适

◯ 中度不适

◯ 稍严重不适

◯ 严重不适

◯ 极其严重不适

*24. 在过去的一周里，您是否被腹泻困扰?

◯ 无不适

◯ 稍微不适

◯ 轻度不适

◯ 中度不适

◯ 稍严重不适

◯ 严重不适

◯ 极其严重不适

*25. 在过去的一周里，您是否被时干时稀的稀溏便困扰?

◯ 无不适

◯ 稍微不适

◯ 轻度不适

◯ 中度不适

◯ 稍严重不适

◯ 严重不适

◯ 极其严重不适

*26. 在过去的一周里，您是否被时干时稀的干结便困扰?

◯ 无不适

◯ 稍微不适

◯ 轻度不适

◯ 中度不适

◯ 稍严重不适

◯ 严重不适

◯ 极其严重不适

*27. 在过去的一周里，您是否被排便急迫感困扰?

◯ 无不适

◯ 稍微不适

◯ 轻度不适

◯ 中度不适

◯ 稍严重不适

◯ 严重不适

◯ 极其严重不适

*28. 在过去的一周里，您是否被排便不尽感困扰?

◯ 无不适

◯ 稍微不适

◯ 轻度不适

◯ 中度不适

◯ 稍严重不适

◯ 严重不适

◯ 极其严重不适

第三部分，饮酒情况（共10项问题）

*29. 在过去一年里，您多长时间喝一次酒

◯ 从不喝酒

◯ 每月1次或数个月1次

◯ 每月2–4次

◯ 每星期2–3次

◯ 每星期4次或以上

*30. 在喝酒的那一天，您一般喝多少白酒？（1两=50ml）

◯ 不喝白酒

◯ 1– 2两

◯ 3–4两

◯ 5– 6两

◯ 7– 8两

◯ 9两以上

*31. 在喝酒的那一天，您一般喝多少罐啤酒？（1罐约330ml）

◯ 不喝啤酒

◯ 1–2 罐

◯ 3–4 罐

◯ 5–6 罐

◯ 7–9 罐

◯ 10 罐以上

*32. 在喝酒的那一天，您一般喝多少红酒？（1杯=150ml）

◯ 不喝红酒

◯ 1–2 杯

◯ 3–4 杯

◯ 5–6 杯

◯ 7–9 杯

◯ 10 杯以上

*33. 您多久会有一次场合，喝酒超过：6罐啤酒，或6两白酒，或6杯红酒

◯ 从来没有

◯ 2个月或数个月1次

◯ 每月1次

◯ 每周1次

◯ 每天1次，或接近每天1次

*34. 在过去一年中，以下情况有多久出现：
发现一旦开始喝酒便无法停止

◯ 从来没有

◯ 2个月或数个月1次

◯ 每月1次

◯ 每周1次

◯ 每天1次，或接近每天1次

*35. 在过去一年中，以下情况有多久出现：
因为喝酒，耽误了计划要做的事情

◯ 从来没有

◯ 2个月或数个月1次

◯ 每月1次

◯ 每周1次

◯ 每天1次，或接近每天1次

*36. 在过去一年中，以下情况有多久出现：
在大量饮酒后，必须喝一顿晨酒（早酒），才能重振精神

◯ 从来没有

◯ 2个月或数个月1次

◯ 每月1次

◯ 每星期1次

◯ 每天1次，或接近每天1次

*37. 在过去一年中，以下情况有多久出现：
在酒后，觉得愧疚或自责

◯ 从来没有

◯ 2个月或数个月1次

- ◯ 每月1次
- ◯ 每星期1次
- ◯ 每天1次，或接近每天1次

*38. 在过去一年中，以下情况有多久出现：
酒后忘记前一晚上发生的事情

- ◯ 从来没有
- ◯ 2个月或数个月1次
- ◯ 每月1次
- ◯ 每星期1次
- ◯ 每天1次，或接近每天1次

*39. 您是否有以下情况：
因为喝酒，伤害过自己或他人

- ◯ 没有
- ◯ 有，但不在近一年中
- ◯ 有，在近一年中

*40. 您是否有以下情况：
您的亲戚、朋友、医生或其他保健工作人员，
有没有关心过您的饮酒问题，或是建议您少喝点?

- ◯ 没有
- ◯ 有，但不在近一年中
- ◯ 有，在近一年中

第四部分，吸烟情况（共13项问题）

*41. 请问您是否吸烟

- ◯ 不吸烟
- ◯ 吸烟

*42. 请给您的烟瘾程度打分（0分表示没有烟瘾，100分表示极度上瘾）

0 ◯ ◯ ◯ ◯ ◯ ◯ ◯ ◯ ◯ ◯ ◯ 100

*43. 您平均每天抽多少支烟?

- ◯ 0–5 支
- ◯ 6–10 支
- ◯ 11–20 支
- ◯ 21–29 支

◯ 30 支以上

*44. 您起床后多久会抽第一支烟?

◯ 0–5 分钟
◯ 6–15 分钟
◯ 16–30 分钟
◯ 31–60分钟
◯ 60 分钟以后

*45. 对您来说，戒烟的困难程度有多少?

◯ 完全不可能
◯ 很困难
◯ 有一定困难
◯ 比较容易
◯ 很容易

*46. 是否同意以下观点:
几个小时不抽烟后，我会有迫切且抑制不住的吸烟冲动

◯ 完全不同意
◯ 有点不同意
◯ 不同意，也不反对
◯ 稍微同意
◯ 完全同意

*47. 是否同意以下观点:
一想到没有香烟，我就会感到压力

◯ 完全不同意
◯ 有点不同意
◯ 不同意，也不反对
◯ 稍微同意
◯ 完全同意

*48. 是否同意以下观点:
出门前，我总是确保随时携带香烟

◯ 完全不同意
◯ 有点不同意
◯ 不同意，也不反对
◯ 稍微同意
◯ 完全同意

*49. 是否同意以下观点：
我成了香烟的俘虏

○ 完全不同意
○ 有点不同意
○ 不同意，也不反对
○ 稍微同意
○ 完全同意

*50. 是否同意以下观点：
我感觉抽烟太多了

○ 完全不同意
○ 有点不同意
○ 不同意，也不反对
○ 稍微同意
○ 完全同意

*51. 是否同意以下观点：
有时我会放下所有的事情出去买烟

○ 完全不同意
○ 有点不同意
○ 不同意，也不反对
○ 稍微同意
○ 完全同意

*52. 是否同意以下观点：
我一直在抽烟

○ 完全不同意
○ 有点不同意
○ 不同意，也不反对
○ 稍微同意
○ 完全同意

*53. 是否同意以下观点：
尽管会对我的身体健康产生危害，但我还是会选择抽烟

○ 完全不同意
○ 有点不同意
○ 不同意，也不反对
○ 稍微同意
○ 完全同意

第五部分，个人压力情况（共10项问题）

请根据第一印象选择

*54. 在过去一个月里，以下事件发生的频率为：
您因意外发生的事情而心烦意乱

- ○ 完全没有
- ○ 几乎没有
- ○ 有时
- ○ 经常
- ○ 大多数时候

*55. 在过去一个月里，以下事件发生的频率为：
您感觉没有能力控制生活中的重要事情

- ○ 完全没有
- ○ 几乎没有
- ○ 有时
- ○ 经常
- ○ 大多数时候

*56. 在过去一个月里，以下事件发生的频率为：
您感到紧张或者压力

- ○ 完全没有
- ○ 几乎没有
- ○ 有时
- ○ 经常
- ○ 大多数时候

*57. 在过去一个月里，以下事件发生的频率为：
您对自己处理个人问题的能力感到信心十足

- ○ 完全没有
- ○ 几乎没有
- ○ 有时
- ○ 经常
- ○ 大多数时候

*58. 在过去一个月里，以下事件发生的频率为：
感觉事情正按照您预定的方向发展

- ○ 完全没有
- ○ 几乎没有
- ○ 有时
- ○ 经常

◯ 大多数时候

*59. 在过去一个月里，以下事件发生的频率为：
感觉无法应付您必须做的事情

◯ 完全没有

◯ 几乎没有

◯ 有时

◯ 经常

◯ 大多数时候

*60. 在过去一个月里，以下事件发生的频率为：
您感觉能控制生活中让人烦恼的事情

◯ 完全没有

◯ 几乎没有

◯ 有时

◯ 经常

◯ 大多数时候

*61. 在过去一个月里，以下事件发生的频率为：
您感觉到自己掌控了一切

◯ 完全没有

◯ 几乎没有

◯ 有时

◯ 经常

◯ 大多数时候

*62. 在过去一个月里，以下事件发生的频率为：
因为发生了一些超出您控制范围的事情而感到愤怒

◯ 完全没有

◯ 几乎没有

◯ 有时

◯ 经常

◯ 大多数时候

*63. 在过去一个月里，以下事件发生的频率为：
感觉到困难堆积如山，以至于您无法克服它们

◯ 完全没有

◯ 几乎没有

◯ 有时

◯ 经常

◯ 大多数时候

第六部分，睡眠情况（共23项问题）
你的答案应该是能代表过去一个月中，大多数日夜的答复

*64. 在过去的一个月里，您通常晚上几点钟上床睡觉?

*65. 在过去的一个月里，您通常需要多少分钟才能入睡？（请填写分钟）

*66. 在过去的一个月里，您通常早上几点钟起床?

*67. 在过去的一个月里，您晚上的实际睡眠时间是几个小时?

*68. 在过去的一个月里，您因为下列原因而导致睡眠不好的频率为：
不能在30分钟内入睡

◯ 在过去的一个月没有出现
◯ 2周或者数周一次
◯ 每周1–2次
◯ 每周3次或以上

*69. 在过去的一个月里，您因为下列原因而导致睡眠不好的频率为：
在半夜或清晨时醒来

◯ 在过去的一个月没有出现
◯ 2周或者数周一次
◯ 每周1–2次
◯ 每周3次或以上

*70. 在过去的一个月里，您因为下列原因而导致睡眠不好的频率为：
睡觉途中不得不起夜上厕所

◯ 在过去的一个月没有出现
◯ 2周或者数周一次
◯ 每周1–2次
◯ 每周3次或以上

*71. 在过去的一个月里，您因为下列原因而导致睡眠不好的频率为：
呼吸不畅

◯ 在过去的一个月没有出现
◯ 2周或者数周一次
◯ 每周1-2次
◯ 每周3次或以上

*72. 在过去的一个月里，您因为下列原因而导致睡眠不好的频率为：
大声咳嗽或打鼾

◯ 在过去的一个月没有出现
◯ 2周或者数周一次
◯ 每周1-2次
◯ 每周3次或以上

*73. 在过去的一个月里，您因为下列原因而导致睡眠不好的频率为：
感觉太冷

◯ 在过去的一个月没有出现
◯ 2周或者数周一次
◯ 每周1-2次
◯ 每周3次或以上

*74. 在过去的一个月里，您因为下列原因而导致睡眠不好的频率为：
感觉太热

◯ 在过去的一个月没有出现
◯ 2周或者数周一次
◯ 每周1-2次
◯ 每周3次或以上

*75. 在过去的一个月里，您因为下列原因而导致睡眠不好的频率为：
噩梦

◯ 在过去的一个月没有出现
◯ 2周或者数周一次
◯ 每周1-2次
◯ 每周3次或以上

*76. 在过去的一个月里，您因为下列原因而导致睡眠不好的频率为：
自觉疼痛

◯ 在过去的一个月没有出现
◯ 2周或者数周一次
◯ 每周1-2次
◯ 每周3次或以上

77. 在过去的一个月里，您有没有上面问题之外的“其他原因”，而导致睡眠不好。
如果有，请描述原因

78. 在过去一个月里，您因为“其他原因”而导致睡眠不好的频率为：

- ◯ 在过去一个月没有出现
- ◯ 2周或数周一次
- ◯ 每周1-2次
- ◯ 每周3次以上

*79. 在过去的一个月里，您对自己的睡眠总体评价如何?

- ◯ 很好
- ◯ 一般
- ◯ 不太好
- ◯ 很差

*80. 在过去一个月里，您多久吃一次药物来帮助您入睡

- ◯ 过去一个月没有出现
- ◯ 2周或者数周1次
- ◯ 每周1-2次
- ◯ 每周3次或以上

*81. 在过去的一个月里，您在开车、吃饭或者参加社交活动，很难保持清醒的频率为

- ◯ 在过去的一个月没有出现
- ◯ 2周或者数周一次
- ◯ 每周1-2次
- ◯ 每周3次或以上

*82. 在过去一个月里，对于您来说，保持足够的热情来完成任务，有多大的问题

- ◯ 完全没有问题
- ◯ 只有很小的问题
- ◯ 有一些问题
- ◯ 有很大问题

*83. 您是否有伴侣或室友

- ◯ 没有伴侣或室友
- ◯ 有伴侣或室友，但在不同房间
- ◯ 伴侣在同一个房间，但不是一张床

◯ 有伴侣，并在同一张床上

*84. 让伴侣 / 室友告诉您，过去一个月里，您出现以下行为的频率是？
响亮的鼾声

◯ 过去一个月没有出现
◯ 2周或数周1次
◯ 每周1–2次
◯ 每周3次或以上

*85. 让伴侣 / 室友告诉您，过去一个月里，您出现以下行为的频率是？
睡觉时，两次呼吸之间长时间停顿

◯ 过去一个月没有出现
◯ 2周或数周1次
◯ 每周1–2次
◯ 每周3次或以上

*86. 让伴侣 / 室友告诉您，过去一个月里，您出现以下行为的频率是？
睡觉时，两腿抽搐或者抽筋

◯ 过去一个月没有出现
◯ 2周或数周1次
◯ 每周1–2次
◯ 每周3次或以上

*87. 让伴侣 / 室友告诉您，过去一个月里，您出现以下行为的频率是？
睡眠时出现迷失方向或神志不清

◯ 过去一个月没有出现
◯ 2周或数周1次
◯ 每周1–2次
◯ 每周3次或以上

第七部分，焦虑情况（共14项问题）
勾选与您过去一周感觉最接近的回复，您的第一感觉是最好的答案

*88. 感到紧张

◯ 大部分时间
◯ 很多时候
◯ 有时、偶尔
◯ 根本没有

*89. 仍然热衷于曾经喜欢的东西

◯ 完全如此

○ 感兴趣，但没有那么热爱
○ 只有一点点兴趣
○ 几乎没有兴趣

*90. 有一种恐惧的预感，好像可怕的事情将要发生

○ 非常肯定，而且是非常糟糕的
○ 是的，但事情不算太糟糕
○ 有这样的预感，但我并不担心
○ 完全没有

*91. 我很乐观，能看到事物有趣的一面

○ 总是这样
○ 是，但不如以前
○ 大不如前
○ 完全没有

*92. 忧心忡忡的想法在脑中徘徊

○ 大部分时间
○ 很多时候
○ 有时
○ 偶尔

*93. 我觉得很开心

○ 一点也不
○ 很少
○ 有时
○ 大多数时候

*94. 可以安心的坐着，感觉很放松

○ 完全如此
○ 经常
○ 偶尔
○ 完全没有

*95. 我觉得我好像变迟钝了

○ 基本上一直是这样
○ 很多时候
○ 有些时候

○ 完全没有

*96. 感到焦虑不安，内心七上八下

○ 完全没有
○ 偶尔
○ 经常
○ 大部分时候

*97. 我不再注重自己的外表

○ 完全如此
○ 很少注重
○ 仍在注重，但比以前少
○ 我一如既往地注重自己的外表

*98. 当我得一直做事时，会感到焦虑不安

○ 确实如此
○ 多数时候
○ 不是很频繁
○ 完全没有

*99. 我期待去享受事物

○ 和以前一样期待
○ 跟过去比相对少一点
○ 大不如前
○ 完全没有

*100. 突然有一种惊慌失措的感觉

○ 大部分时候
○ 很多时候
○ 偶尔
○ 完全没有

*101. 我会享受阅读一本好书，或听收音机，或看电视节目

○ 经常
○ 有时
○ 偶尔
○ 很少

提交

问卷星 提供技术支持

举报
